# *Faecalibacterium prausnitzii* alleviates inflammatory arthritis and regulates IL-17 production, short chain fatty acids, and the intestinal microbial flora in experimental mouse model for rheumatoid arthritis

**DOI:** 10.1186/s13075-023-03118-3

**Published:** 2023-07-26

**Authors:** Jeonghyeon Moon, A. Ram Lee, Heejung Kim, JooYeon Jhun, Seon-Yeong Lee, Jeong Won Choi, Yunju Jeong, Myeong Soo Park, Geun Eog Ji, Mi-La Cho, Sung-Hwan Park

**Affiliations:** 1 Departments of Neurology and Immunobiology, Yale School of Medicine, New Haven, CT USA; 2 Department of Biomedicine & Health Sciences, College of Medicine, The Catholic University of Korea, Seoul, 06591 Republic of Korea; 3 Rheumatism Research Center, College of Medicine, Catholic Research Institute of Medical Science, The Catholic University of Korea, Seoul, 06591 Korea; 4 Lab of Translational ImmunoMedicine, Catholic Research Institute of Medical Science, College of Medicine, The Catholic University of Korea, 222, Banpo-Daero, Seocho-Gu, Seoul, 06591 Republic of Korea; 5 Department of Food and Nutrition, Research Institute of Human Ecology, Seoul National University, Seoul, South Korea; 6 Harvard Medical School, Boston, MA USA; 7 Division of Pulmonary and Critical Care Medicine, Brigham and Women’s Hospital, Boston, MA USA; 8 Research Center, BIFIDO Co., Ltd., Hongcheon, South Korea; 9 Department of Medical Life Sciences, College of Medicine, The Catholic University of Korea, 222, Banpo-Daero, Seocho-Gu, Seoul, 06591 Republic of Korea; 10 Division of Rheumatology, Department of Internal Medicine, College of Medicine, Seoul St. Mary’s Hospital, The Catholic University of Korea, Seoul, Korea

**Keywords:** Rheumatoid arthritis, *Faecalibacterium prausnitzii*, Interleukin-17, Microbial flora, Short-chain fatty acids

## Abstract

**Background:**

Rheumatoid arthritis (RA) is a systemic chronic inflammatory disease that leads to joint destruction and functional disability due to the targeting of self-antigens present in the synovium, cartilage, and bone. RA is caused by a number of complex factors, including genetics, environment, dietary habits, and altered intestinal microbial flora. Microorganisms in the gut bind to nod-like receptors and Toll-like receptors to regulate the immune system and produce various metabolites, such as short-chain fatty acids (SCFAs) that interact directly with the host. *Faecalibacterium prausnitzii* is a representative bacterium that produces butyrate, a well-known immunomodulatory agent in the body, and this microbe exerts anti-inflammatory effects in autoimmune diseases.

**Methods:**

In this study, *F. prausnitzii* was administered in a mouse model of RA, to investigate RA pathology and changes in the intestinal microbial flora. Using collagen-induced arthritic mice, which is a representative animal model of RA, we administered *F. prausnitzii* orally for 7 weeks.

**Results:**

The arthritis score and joint tissue damage were decreased in the mice administered *F. prausnitzii* compared with the vehicle-treated group. In addition, administration of *F. prausnitzii* reduced the abundance of systemic immune cells that secrete the pro-inflammatory cytokine IL-17 and induced changes in SCFA concentrations and the intestinal microbial flora composition. It also resulted in decreased lactate and acetate concentrations, an increased butyrate concentration, and altered compositions of bacteria known to exacerbate or improve RA.

**Conclusion:**

These results suggest that *F. prausnitzii* exerts a therapeutic effect on RA by regulation of IL-17 producing cells. In addition, *F. prausnitzii* modify the microbial flora composition and short chain fatty acids in experimental RA mouse model.

## Background

Rheumatoid arthritis (RA) is a systemic inflammatory autoimmune disease characterized by inflammation and the proliferation of synovial cells, as well as osteoporosis and erosion of bone and joints [1, 2]. Various types of T lymphocytes, such as activated T helper (Th) 1, Th17, and autoreactive CD4^+^ T cells, and the secretion of pro-inflammatory cytokines such as interleukin (IL)-17, IL-23, IL-6, and TNF-α, are mainly responsible for the development of RA [3–6].

Although many targeted treatments have been developed for RA alleviation, none can achieve a complete cure because the pathogenesis of RA is not fully understood [7, 8]. Numerous genetic and environmental factors are associated with an increased risk of RA [9]. Factors such as smoking, hormones, microorganisms, and infection may be involved in RA induction [10–12].

Dysbiosis of the commensal microbiota is among the most important factors associated with RA development and progression [13–15]. In cooperation with intestinal lymphoid tissue, the intestinal microbiota helps maintain immune homeostasis and serves as a marker of the host’s health status [16]. For example, bacterial cells can pass through mucus and stimulate epithelial cells to regulate immune function and enhance the intestinal barrier [17]. The intestinal microbiota can also interact directly with the host via microbial metabolites, such as short-chain fatty acids (SCFAs), to regulate the immune system [18, 19].

*Faecalibacterium prausnitzii* (formerly known as *Fusobacterium prausnitzii*) is an anaerobic gram-positive, non-spore-forming, acetate-consuming, butyrate-producing, and extremely oxygen-sensitive bacterium [20, 21]. *F. prausnitzii* is less abundant in diseased patients with inflammatory diseases such as RA [22], Sjögren’s syndrome [23], and multiple sclerosis [24], whereas it comprises 5–15% of the gut bacteria in healthy adults, suggesting that the relative distribution of *F. prausnitzii* is a key biomarker of disease [25, 26].

The anti-inflammatory effect of *F. prausnitzii*, which is well known in Crohn’s disease [27], asthma [28], and multiple sclerosis [29], also supports the importance of *F. prausnitzii* in various inflammatory diseases [30]. As a butyrate producer, the anti-inflammatory effect of *F. prausnitzii* has been attributed to butyrate production [31, 32]. Specifically, *F. prausnitzii* attenuates colitis by producing butyrate to reduce Th17 cell differentiation, and inhibit NF-κB activation and histone deacetylase (HDAC) 3 expression in T cells [33, 34]. The supernatant from *F. prausnitzii* cells attenuated colitis of mice model and in vitro human cells by inhibiting the differentiation of pro-inflammatory Th17 cells [35]. In addition, *F. prausnitzii*-derived butyrate ameliorated colitis via HDAC3 inhibition [36]. Butyrate relieved inflammatory arthritis in a collagen-induced arthritis (CIA) mouse model by inhibiting HDAC2 in osteoclasts and HDAC8 in T cells [37]. However, the mechanism of action of *F. prausnitzii* in RA is still unknown. In this study, we show that the anti-inflammatory effect of *F. prausnitzii* does not appear to be limited to butyrate.

We investigated the anti-inflammatory and immune-cell-modulatory effects, SCFA levels, and intestinal microbial flora changes induced by *F. prausnitzii* in a CIA mouse model, which is the most widely used animal model in RA research.

## Materials and methods

### Preparation of animals and *F. prausnitzii*

All animal experimental designs and procedures were approved by the animal research ethics committee of the Catholic University of Korea (approval number CUMC-2019–0242-01). Male DBA/1 J mice (Orient Bio, Seongnam, Korea) were maintained in a specific pathogen-free, temperature-controlled environment (22 ± 2 °C) at a relative humidity of 50 ± 10% under a 12 h light/dark cycle. The mice had ad libitum access to standard mouse chow (Ralston Purina, Gray Summit, MO, USA) and water. The fecal isolates *F. prausnitzii* A2-165 was used. Commercial strains of *F. prausnitzii* A2-165 was purchased from DSMZ-German Collection of Microorganisms and Cell Cultures (DSMZ, Braunschweig, Germany). *F. prausnitzii* A2-165 were cultured anaerobically for 24 h at 37 °C in modified reinforced clostridial (MRC) broth in Hungate tubes. The bacteria were harvested by centrifugation at 8000 × g for 10 min at 4 °C, washed in phosphate-buffered saline, and re-suspended in sterile saline. The cells were administered orally (1 × 10^9^ CFU) in 200 μL sterile saline. To assess the anti-inflammatory effect of *F. prausnitzii* on the development of RA, 10-week-old DBA/1 J mice were orally administered *F. prausnitzii* (Faecali group) or sterile saline (Vehicle group) for 7 weeks after observation of RA symptoms induced by collagen injection.

### Induction of arthritis and *F. prausnitzii* administration

DBA/1 J mice (6–7 weeks old) were randomly divided into three groups (*n* = 5 per group): Faecali group, Vehicle group, and Heat-killed Faecali group. Mice were immunized with 100 µg chicken type II collagen (Chondrex Inc., Redmond, WA, USA) dissolved overnight in 0.1 N acetic acid (4 mg/mL) in complete Freund’s adjuvant. The immunizations were given intradermally via the base of the tail. Two weeks after the primary immunization, mice were boosted with 100 µg type II collagen in incomplete Freund’s adjuvant (Chondrex Inc., Redmond, WA, USA). CIA mice were administered 10^9^ CFU *F. prausnitzii* daily, in a sterile saline suspension in a total volume of 200 µl.

### Clinical scoring of arthritis

The onset, duration, and severity of joint inflammation were evaluated twice weekly for 10 weeks after the primary immunization. Arthritis severity was scored using the mean arthritis index on a scale of 0–4, as follows: 0, no swelling; 1, mild swelling confined to the toes; 2, erythema and mild swelling extending from the ankle to the midfoot; 3, moderate swelling extending from the ankle to the metatarsal joints; and 4, severe swelling encompassing the ankle, foot, and digits. Arthritis severity was determined as the sum of the scores for all legs, as assessed by two independent observers blinded to the experimental groups.

### Histopathological analysis

Histological analysis was performed to determine the extent of joint damage. The joint tissues of mice were fixed in 4% paraformaldehyde, decalcified in a histological decalcifying agent (Calci-Clear Rapid; National Diagnostics, Atlanta, GA, USA), embedded in paraffin, and sectioned. The sections were deparaffinized using xylene and dehydrated using an alcohol gradient. Sections were stained with hematoxylin and eosin and safranin O.

### Immunohistochemical analysis

Paraffin-embedded sections were incubated at 4 °C with the following primary monoclonal antibodies: anti-IL-1β (ab9722; Abcam, Cambridge, UK), anti-TNF-α (ab6671; Abcam), and anti-IL-17 (ab79056; Abcam). Then, the samples were incubated with the respective secondary biotinylated antibodies, followed by incubation for 30 min with streptavidin–peroxidase complex. The reaction product was developed using 3,3-diaminobenzidine chromogen (K3468; Dako, Santa Clara, CA, USA).

### Flow cytometry

The cells used for analysis of the CD4^+^ IFNγ ^+^ (Th1), CD4^+^ IL-4^+^ (Th2), CD4^+^ IL-17^+^ (Th17), and IL-17-secreting CD19^+^ B cell (B17) populations were stimulated with PMA and ionomycin for 4 h using GolgiStop (BD Biosciences, Franklin Lake, NJ, USA). To quantify the Th1, Th2, Th17, and B17 cell populations, mouse splenocytes were immunostained using a PerCp5.5-conjugated anti-CD4 antibody (eBioscience, San Diego, CA, USA) for T cells and a PerCP5.5-conjugated anti-CD19 antibody for B cells. The cells were fixed and permeabilized using the Cytofix/Cytoperm Plus kit (BD Biosciences) following the manufacturer’s instructions, and then stained with APC-conjugated anti-IFNγ, PE-conjugated anti-IL-4, APC-conjugated anti-IL-10, and FITC-conjugated anti-IL-17 antibodies (eBioscience), respectively. All samples were analyzed using the Attune NxT Flow cytometer (Thermo Fisher Scientific, Waltham, MA, USA). The flow cytometry data were analyzed using FlowJo™ software (FlowJo, Ashland, OR, USA).

### Analysis of short-chain fatty acids

The cecum and serum samples were homogenized in 400 µl DW and then acidified using 25% metaphosphoric acid (Sigma Aldrich, St. Louis, MO, USA), at a 1:5 ratio of acid to sample, on ice for 30 min. The samples were centrifuged at 15,000 × g for 15 min, and the supernatants were stored at − 80 °C until further processing. SCFAs such as acetate, lactate, and butyrate were analyzed by high-performance liquid chromatography, using the YL9100 system (Young-lin, Anyang, Korea) equipped with a refractive index detector and Aminex HPX-87H column (300 × 7.8 mm; Bio-Rad Laboratories, Hercules, CA, USA). The liquid culture medium was centrifuged at 18,000 × g for 15 min, and the supernatant was filtered through a 0.2-µm filter. For the analysis, 5 mM sulfuric acid (J.T. Baker, Phillipsburg, MA, USA) was used as the mobile phase, with a flow rate of 0.6 mL/min. The concentration of each organic acid (mM) was normalized to the corresponding concentration of the external SCFA standard.

### DNA extraction and 16S rRNA gene amplification and sequencing

DNA from cecum samples (100 mg) was extracted using the QIAamp Fast DNA Mini Kit (Qiagen, Hilden, Germany) according to the manufacturer’s instructions. The V3–V4 region of the bacterial 16S rRNA gene was amplified using 16S amplicon PCR forward (5′‑TCG TCG GCA GCG TCA GAT GTG TAT AAG AGA CAG CCT ACG GGN GGC WGC AG‑3′) and reverse (5′‑GTC TCG TGG GCT CGG AGA TGT GTA TAA GAG ACA GGA CTA CHV GGG TAT CTA ATC C‑3′) primers. The resulting products were purified, quantified, and pooled together at equal concentrations (5 ng/µl), as described previously [38]. The purified products were sequenced using the MiSeq platform (Illumina, San Diego, CA, USA).

### Bioinformatic analysis

Data analysis was performed using Quantitative Insights Into Microbial Ecology 2 (version 2020.3; http://qiime2.org), as described previously [38]. Paired–end sequences were joined using fastq-join, demultiplexed, and quality-filtered. The filter parameters for trimming and truncating using the DADA2 plugin were 0 and 280, respectively, to remove low-quality sequence regions. Chimeric sequences were removed, and reads were clustered into operational taxonomic units (OTUs) using the Greengenes 13_8 database, with a 99% sequence identity cutoff. Analyses were performed using a rarefied table of 11,000 sequences per sample. In addition, beta diversity was analyzed using the Bray–Curtis, Jaccard, and UniFrac indexes. Differential OTUs among groups and cladograms were identified based on the linear discriminant analysis effect size (LEfSe).

### Statistical analysis

Cecal and serum SCFA data were compared by ordinary one-way ANOVA, and Dunnett’s multiple comparison test was used to compare the experimental and control groups. The number of reads from the feature/OTU table was normalized using the cumulative sum method to generate relative abundances. Non-parametric statistical analyses, including the Kruskal–Wallis test, were performed to compare the relative abundances of taxa and alpha diversity in each group. All statistical analyses were performed using Prism 8 (GraphPad Software, Inc., San Diego, CA, USA).

## Results

### *F. prausnitzii* attenuates the progression of RA in CIA mice

**Fig. 1:**
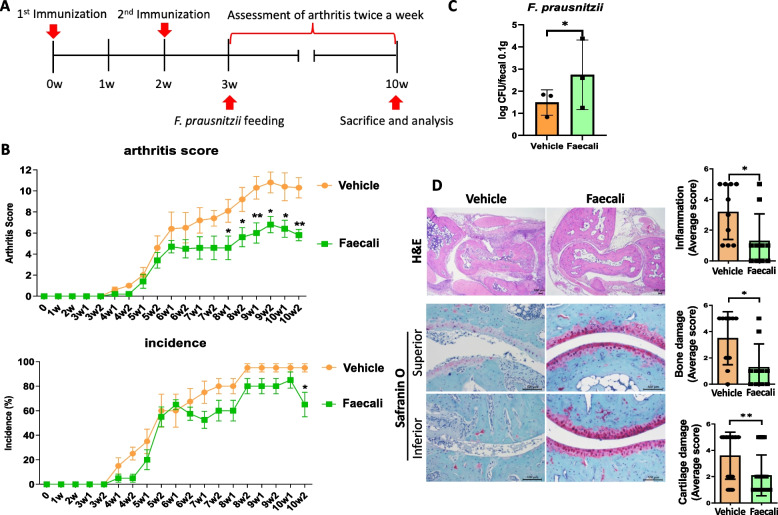
RA pathogenesis is prevented by oral administration of *F. prausnitzii* in CIA mice. **A** Timeline of the immunization and *F. prausnitzii* administration procedure. **B** The arthritis clinical score and incidence were reduced after oral *F. prausnitzii* administration in CIA mice. Arthritis was observed twice a week from week 3 to week 10, which is indicated on the *X*-axis (w = week). **C** Mice were sacrificed after 10 weeks *F. prausnitzii* administration and feces were collected. Quantitative PCR results showing elevated *F. prausnitzii* abundance in the feces of *F. prausnitzii-*treated mice. **D** Microscopy images of histologic sections of hind leg joints of Vehicle and Faecali group mice stained with hematoxylin and eosin (H&E) and safranin O. H&E images were displayed at × 40 magnification and safranin O images at × 200 magnification, respectively. The data are means ± standard deviation (*n* = 5). *p < 0.05 and **p < 0.01

To investigate the anti-inflammatory effects of *F. prausnitzii*, we induced arthritis in mice via two immunizations at 2-week intervals. Starting at 1 week after the second immunization, 10^9^ CFU *F. prausnitzii* (Faecali group) and saline (Vehicle group) were administered to the mice, daily for 7 weeks (Fig. 1A). The arthritis score was decreased significantly at week 8, and the incidence of arthritis was notably decreased at week 10, in Faecali mice (Fig. 1B). After 10 weeks of *F. prausnitzii* administration, the mice were sacrificed and analyzed. Quantitative PCR was performed to detect *F. prausnitzii* in mouse feces, and the results showed significantly increased abundance of *F. prausnitzii* in the Faecali group (Fig. 1C). The level of damage in joint tissue was confirmed by hematoxylin and eosin and safranin O staining. Bone erosion and cartilage damage were significantly reduced in the Faecali group compared with the Vehicle group (Fig. 1D). These results suggest that administration of *F. prausnitzii* prevents arthritis progression and damage to joint tissues.

### *F. prausnitzii* regulates systemic immune cell populations and inhibits IL-17 secretion 

**Fig. 2:**
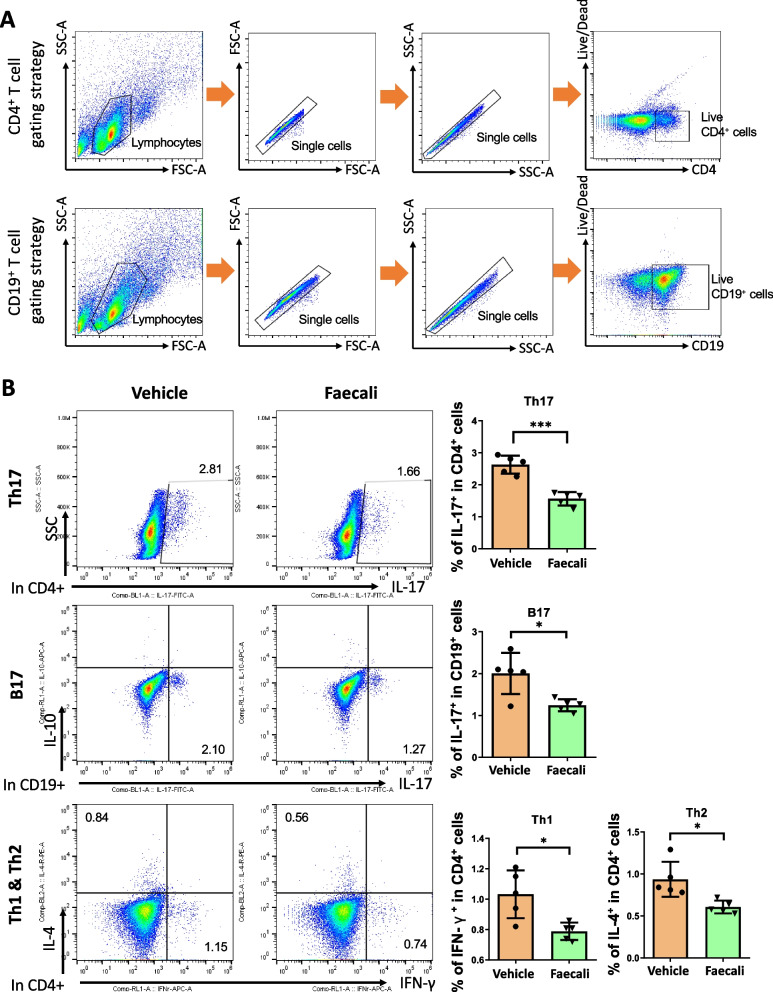
Immunomodulatory effect of *F. prausnitzii* administration on mouse splenocytes. Th17 (CD4^+^ IL-17^+^), B17 (CD19^+^ IL-17^+^), and Th1 (CD4^+^ IFN-γ^+^)/Th2 (CD4^+^ IL-4^+^) cell populations were analyzed by flow cytometry. The data are means ± standard deviation (*n* = 5). * *p* < 0.05 and *** *p* < 0.001

As systemic immune cells are closely involved in the progression of autoimmune arthritis and tissue damage, we stained mouse splenocytes and analyzed systemic immune cells by flow cytometry. The proportion of IL-17^+^ cells among CD4^+^ cells (Th17) was decreased in the Faecali group. Among CD19^+^ cells, the proportion of IL-17-secreting cells (B17) was significantly reduced in the Faecali group, while there was no significant change in the abundance of IL-10-secreting cells (data not shown). The abundances of other subtypes of helper T cells, i.e., CD4^+^ IFNγ^+^ (Th1) and CD4^+^ IL-4^+^ (Th2) cells, were also decreased by *F. prausnitzii* administration (Fig. 2). These results suggest that *F. prausnitzii* inhibits IL-17 secretion in both CD4^+^ and CD19^+^ cells and induces an anti-inflammatory effect by regulating systemic immune cell populations.

### *F. prausnitzii* decreases pro-inflammatory cytokine levels in joint tissues

**Fig. 3:**
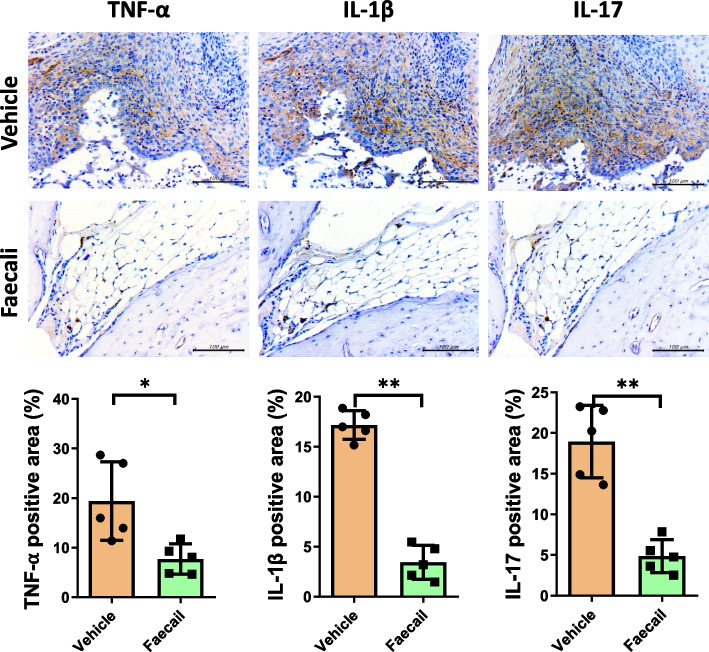
Administration of *F. prausnitzii* leads to a decrease in pro-inflammatory cytokine levels in mouse joint tissues. The levels of TNF-α, IL-1β, and IL-17, which are pro-inflammatory cytokines, were evaluated by immunohistochemistry. The data are means ± standard deviation (*n* = 5). *p < 0.05 and **p < 0.01

To identify the local inflammatory response and immune cell infiltration, we performed immunohistochemical analysis of the pro-inflammatory cytokines TNF-α, IL-1β, and IL-17 in mouse synovial tissue. The levels of TNF-α, IL-1β, and IL-17 were decreased in the Faecali group. In addition, the Faecali group mice were similar to normal mice in term of adipose tissue. In the Vehicle group, fibrosis with immune cell infiltration was observed (Fig. 3). These results suggest that *F. prausnitzii* reduces immune cell infiltration, pro-inflammatory cytokine expression and, by extension, fibrosis in joint tissue.

### *F. prausnitzii* regulates SCFA concentrations and the phylum-level microbial flora

**Fig. 4:**
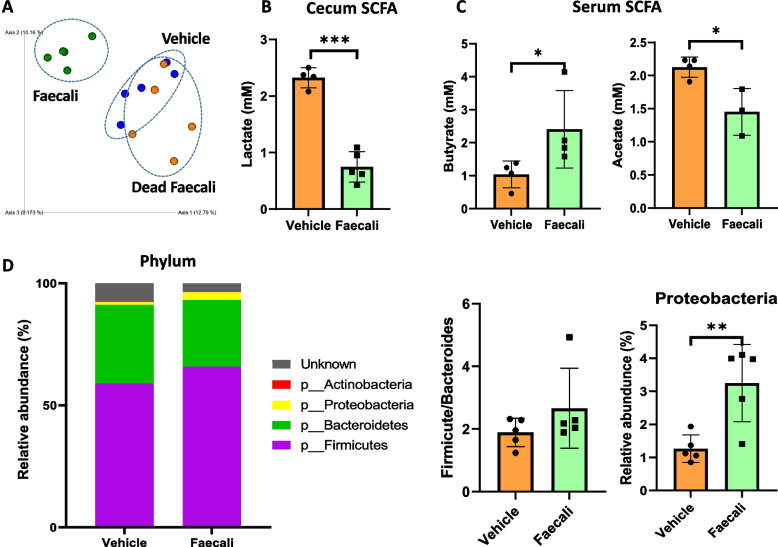
SCFA concentrations and fecal microbial abundances in mice with RA. **A** Effect of *F. prausnitzii* administration on bacterial beta diversity based on the Bray–Curtis distance in mice with RA. **B** The concentrations of SCFAs in cecum (lactate) and **C** serum (butyrate and acetate). **D** The average relative abundances of phyla in the Vehicle and Faecali groups. The data are means ± standard deviation (*n* = 5). *p < 0.05, **p < 0.01, and ***p < 0.001

To determine the effect of *F. prausnitzii* administration on the microbial flora of CIA mice, blood was drawn by retro-orbital blood collection immediately before sacrifice after the end of the experiment. Cecal samples were collected after sacrifice. We also injected heat-killed *F. prausnitzii* as a positive control for *F. prausnitzii* administration (data not shown). In the mice administered heat-killed *F. prausnitzii*, there was a slight reduction in the arthritis score but no significant changes in the SCFA concentrations or intestinal microbial flora. We evaluated beta diversity using the Bray–Curtis index. The Vehicle and positive control groups had similar Bray–Curtis index values, which were different from that of the Faecali group (Fig. 4A). SCFA concentrations were measured in the cecum and serum. The lactate level in the cecum was significantly decreased (Fig. 4B), and the butyrate and acetate levels in serum were increased and decreased, respectively, by *F. prausnitzii* administration (Fig. 4C). Next, we investigated whether *F. prausnitzii* affects the microbial flora composition at the phylum level. The abundances of Firmicutes and Bacteroides, which accounted for the majority of the phyla, were increased and decreased, albeit not significantly, by *F. prausnitzii* treatment, respectively. The abundance of *Proteobacteria* was notably elevated in the Faecali group (Fig. 4D). These results suggest that *F. prausnitzii* administration leads to changes in SCFA and intestinal microbial flora compositions.

### *F. prausnitzii* modifies the RA-related intestinal microbial flora at the genus level

**Fig. 5:**
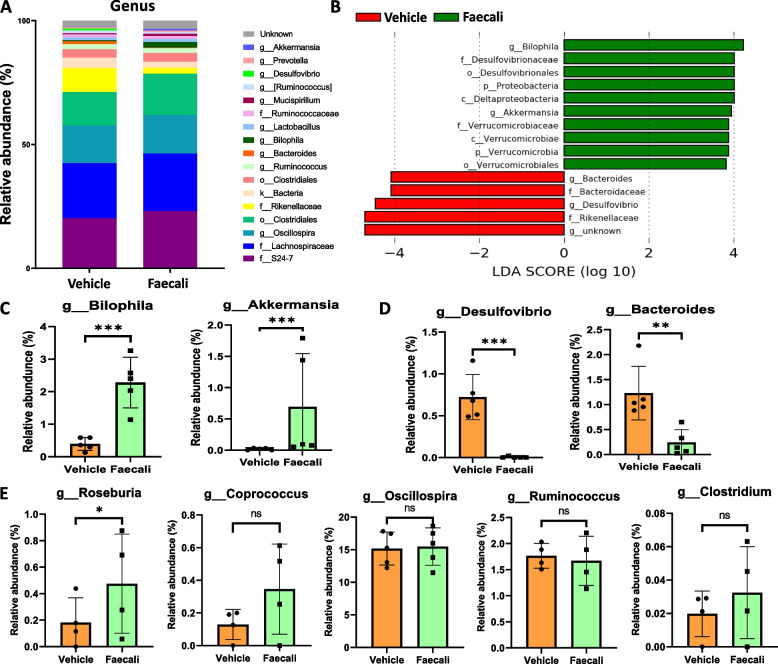
Effect of *F. prausnitzii* administration on the fecal microbial composition at the genus level. **A** The average relative abundances of microbial genera after *F. prausnitzii* administration (taxonomic analysis with relative abundance > 0.5%). **B** Significantly different taxon abundances between the Faecali (green) and Vehicle (red) groups identified by LEfSe analysis (threshold > 2.0). **C** The relative abundances of the genera *Bilophila* and **D** *Akkermansia* and *Desulfovibrio* and *Bacteroides*. **E** The relative abundances of the genera of *Roseburia*, *Coprococcus*, *Oscillospira*, *Ruminococcus*, and *Clostridium* were presented. The data are means ± standard deviation (*n* = 5). *p* < 0.05, **p < 0.01, and ***p < 0.001

To determine whether *F. prausnitzii* administration prevents the progression of RA and inhibits the production of IL-17 via modification of the microbiota, we investigated changes in the microbial flora at the genus level (Fig. 5A). LEfSe analysis is used to identify high-dimensional biomarkers. The contributions of microbial flora changes to the differences between the groups were assessed using the linear discriminant analysis score. LEfSe analysis revealed the top 10 most abundant taxa in the Faecali group and the top 5 most abundant in the Vehicle group (Fig. 5B). Based on LEfSe analysis, the abundances of *Bilophila* and *Akkermansia*, which are negatively associated with pro-inflammatory cytokines production, were increased in the Faecali group (Fig. 5C), while those of *Desulfovibrio* and *Bacteroides*, which are positively associated with pro-inflammatory cytokines secretion, were decreased in the Faecali group (Fig. 5D) [39]. In addition, we investigated whether *F. prausnitzii* administration affects the abundances of other butyrate-producing bacteria, i.e., *Roseburia*, *Coprococcus*, *Oscillospira*, *Ruminococcus*, and *Clostridium*. The abundances of butyrate-producing bacteria tended to be increased by *F. prausnitzii* administration, but only that of *Roseburia* was significantly increased. These data demonstrated that *F. prausnitzii* administration exerts a therapeutic effect on RA by modifying the composition of the intestinal microbial flora (Fig. 5E).

## Discussion

The CIA mouse model, created by injecting DBA/1 J mice with an emulsion of complete Freund’s adjuvant and type II collagen, which induces arthritis in 80–100% of animals, is commonly used to evaluate RA [40, 41]. The CIA mouse model shares immunological and pathological features with human RA [42] and is suitable for investigating the pathogenesis and mechanisms of RA [43]. In this study, we used the CIA mouse model to investigate the protective effects of *F. prausnitzii* on inflammation and the composition of the intestinal microbial flora.

The mechanism of RA is complex and diverse; it involves the genetic background, environment, and microbiome, and complete cure has not yet been achieved [44, 45]. Previous studies showed that butyrate has powerful anti-inflammatory effects in autoimmune disease model mice or human [37, 46, 47]. Hence, this study investigated the global effect of the administration of *F. prausnitzii*, which is a well-known butyrate producer, and the underlying mechanism.

We found that the arthritis score, arthritis incidence, and tissue damage were decreased by *F. prausnitzii* administration. Also, the proportions of IL-17-secreting cells, including Th17 and B17 cells in the spleen, were significantly decreased in the Faecali group compared with the Vehicle group. This result was consistent with a previous study using a DSS-induced colitis mouse model, in which the plasma IL-17A level and percentage of Th17 splenocytes were significantly reduced in the mice treated with *F. prausnitzii* supernatant [35]. Our previous studies reported that butyrate inhibits RA progression by regulating HDAC 8 in T cells and suppresses Sjögren’s syndrome by regulating IL-10-secreting B cells [37, 46]. In addition, Th1 and Th2 cells were reduced in the Faecali group in this study. Immunohistochemistry showed that fibrosis in joint tissue and pro-inflammatory cytokine expression were decreased in the Faecali group compared with the Vehicle group. These data suggests that *F. prausnitzii* may inhibit arthritis in a CIA model by regulating the function of inflammatory immune cells.

We conducted additional experiments to determine how *F. prausnitzii* induces changes in the gut microbial flora and SCFA production. Analysis of the gut microbiota revealed different remodeling patterns among the treatment groups. In the cecum, the lactate level was decreased in the Faecali group. Lactate promotes chronic inflammation and exacerbates RA pathology. SCFAs are the major end products of bacterial fermentation in the colon [48]. Bacterial strains break down complex polysaccharides into monosaccharides, which are further fermented into SCFAs, including acetate, butyrate, and propionate [49, 50]. Butyrate is the key energy source for colonocytes, and plays important roles in the maintenance of colonic mucosal health, inhibition of inflammation and oxidative stress, improvement of barrier function, and promotion of satiety [51]. The serum butyrate level was higher in our Faecali than Vehicle group; however, there was no difference in the cecal butyrate level between the groups (data not shown). In this study, the propionate level was not significantly different between the Faecali and Vehicle groups (data not shown). However, the acetate level was decreased, and the butyrate level increased, in the serum of *F. prausnitzii-*treated mice. *F. prausnitzii* consumes acetate and produces butyrate, and butyrate promotes the differentiation of regulatory T cells [52, 53]. However, in this study, the elevated butyrate level induced by *F. prausnitzii* treatment modulated the abundance of IL-17-secreting immune cells but did not lead to a significant increase in regulatory T cell abundance. This suggests that oral administration of *F. prausnitzii* regulates the composition of SCFAs in the body. In addition, the lactate level in the cecum was decreased by *F. prausnitzii* administration. Lactate activates the tricarboxylic acid cycle via pyruvate in T cells [54]. The resulting fatty acid synthesis not only reduces cell motility but also promotes activation of the transcription factor STAT3 [55]. Activated STAT3 is a well-known inducer of RORγt, which promotes IL-17 production [56, 57].

Interestingly, administration of a single *F. prausnitzii* bacterium elicited changes in the intestinal microbial flora in the RA mouse model. The ratio of Firmicutes to Bacteroidetes, the dominant microbial communities at the phylum level, tended to be elevated in the Faecali group, but the differences between the groups were not significant. Proteobacteria, which has a low abundance in the microbiota, was increased in the Faecali group. Previous studies showed that the Firmicutes to Bacteroidetes ratio and Proteobacteria abundance were decreased in RA patients compared with healthy controls in East Asia [58]. These results suggest that *F. prausnitzii* administration improves the health of the RA mouse model.

In the Faecali group, the abundances of *Bilophila* and *Akkermansia*, which promote RA, were increased*.* The abundance of *Bilophila* is negatively correlated with TNF-α production [39]. The abundance of *Akkermansia muciniphila*, a mucus-colonizing member of microbiota, was increased by *F. prausnitzii* treatment. The mucin degradation activity of *Akkermansia muciniphila* leads to the production of propionate and acetate [59, 60]. On the other hand, *Desulfovibrio* and *Bacteroides* abundances were reduced by *F. prausnitzii* administration. *Desulfovibrio spp*. are anaerobic sulfate-reducing bacteria that colonize the human gut; they are associated with gastrointestinal diseases and stimulation of the epithelial immune response, including nitric oxide production in macrophages [61]. In addition, *Bacteroides fragilis* leads to arthritis and is present in autoimmune disease patients [62–64]. Although the relationship between RA and the intestinal microbial flora is not well understood, this study showed that the administration of *F. prausnitzii* can induce positive changes in the microbial flora for RA.

In summary, this is the first study to evaluate the effects of *F. prausnitzii* on RA in an animal model. *F. prausnitzii* altered gut microbial composition and exerted an anti-inflammatory effect associated with the regulation of IL-17-producing immune cells.

## Conclusion

The aim of this study was to investigate the effect of *F. prausnitzii* administration on SCFA metabolism, immunomodulatory activity, and gut microbial composition. The oral administration of *F. prausnitzii* prevents disease progression and regulates SCFA, proportion of immune cells and intestinal microbial flora on RA mouse model. Hence, this study suggests that *F. prausnitzii* administration is a novel strategy for RA treatment and symptom relief, although it is difficult to cultivate because of its extreme oxygen sensitivity.

## Data Availability

All data are available in the manuscript or upon request to the authors.

## Abbreviations

RA: Rheumatoid arthritis
SCFAs: Short-chain fatty acids
Th: T helper
IL: Interleukin
HDAC: Histone deacetylase
CIA: Collagen-induced arthritis
MRC: Modified reinforced clostridial
IFN: Interferon
OTU: Operational taxonomic units
TNF: Tumor necrosis factor
